# Impact of atherosclerosis on the postoperative complications of colorectal surgery in older patients with colorectal cancer

**DOI:** 10.1186/s12876-022-02600-7

**Published:** 2022-12-14

**Authors:** Takahiro Gunji, Koichi Tomita, Itsuki Koganezawa, Masashi Nakagawa, Kei Yokozuka, Shigeto Ochiai, Toshimichi Kobayashi, Toru Sano, Satoshi Tabuchi, Naokazu Chiba, Eiji Hidaka, Shigeyuki Kawachi

**Affiliations:** Department of Digestive and Transplantation Surgery, Tokyo Medical University Hachioji Medical Center, 1163 Tatemachi, Hachioji-Shi, Tokyo, 193-0998 Japan

**Keywords:** Atherosclerosis, Colorectal cancer, Colorectal surgery, Postoperative complications

## Abstract

**Background:**

Atherosclerosis is associated with various comorbidities; nonetheless, its effect on the postoperative complications of colorectal surgery in older patients with colorectal cancer (CRC) remains unclear. This study aimed to evaluate the impact of atherosclerosis on the postoperative complications of colorectal surgery in older adults with CRC.

**Methods:**

Patients aged ≥ 65 years who underwent surgery for CRC between April 2017 and October 2020 were enrolled. To evaluate atherosclerosis, we prospectively calculated the cardio-ankle vascular index (CAVI) measured by the blood pressure/pulse wave test and abdominal aortic calcification (AAC) score from computed tomography. Risk factors for Clavien–Dindo grade ≥ III postoperative complications were evaluated by univariate and logistic regression analyses.

**Results:**

Overall, 124 patients were included. The mean CAVI value and AAC score were 9.5 ± 1.8 and 7.0 ± 8.0, respectively. Clavien–Dindo grade ≥ III postoperative complications were observed in 14 patients (11.3%). CAVI (odds ratio, 1.522 [95% confidence interval, 1.073–2.160], *p* = 0.019), AAC score (1.083 [1.009–1.163], *p* = 0.026); and operative time (1.007 [1.003–1.012], *p* = 0.001) were identified as risk factors for postoperative complications. Based on the optimal cut-off values of CAVI and AAC score, the probability of postoperative complications was 27.8% in patients with abnormal values for both parameters, which was 17.4 times higher than the 1.6% probability of postoperative complications in patients with normal values.

**Conclusions:**

Atherosclerosis, particularly that assessed using CAVI and AAC score, could be a significant predictor of postoperative complications of colorectal surgery in older adults with CRC.

**Supplementary Information:**

The online version contains supplementary material available at 10.1186/s12876-022-02600-7.

## Background

The proportion of older adults in the society is rapidly increasing, and the share of the global population aged 65 years is projected to rise from 10 in 2022 to 16% in 2050 [1]. Similarly, the proportion of older patients with colorectal cancer (CRC) has been increasing yearly because of the aging population and eating habits [2, 3]. Generally, older patients have more comorbidities that are recognized as risk factors for postoperative complications [4–6]; therefore, they have a higher incidence of postoperative complications than younger patients [7].

Atherosclerosis is one of the crucial pathophysiological processes implicated in cardiovascular diseases, including coronary artery disease, stroke, and heart failure [8, 9], and is increasingly prevalent in older adults with comorbidities such as hypertension, hyperlipidemia, and diabetes mellitus. Previously, visceral fat [10–12], hypoalbuminemia [13], and the prognostic nutritional index [14] were shown to be related to the postoperative outcomes of colorectal surgery. In addition, some recent reports have shown that atherosclerosis is associated with anastomotic leakage after abdominal surgery [15, 16]. Atherosclerosis progresses throughout the body; hence, it is involved in the blood flow of tissues and may cause tissue ischemia, which is the main reason for anastomotic leakage according to previous reports [15, 16]. However, the role of atherosclerosis in colorectal surgery, especially in older patients with CRC, has not been fully investigated previously. It may lead to not only anastomotic leakage but also other postoperative complications.

The present study aimed to evaluate the impact of atherosclerosis on the postoperative complications of colorectal surgery in older patients with CRC using the blood pressure/pulse wave test and scoring of arterial calcifications with computed tomography (CT) scans.

## Methods

### Patients

**Fig. 1:**
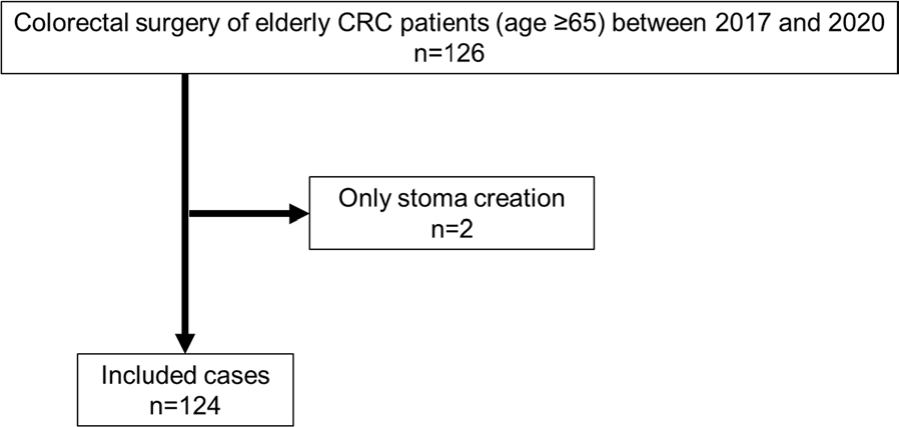
Flowchart of the study

We prospectively collected the clinical data of 126 patients aged ≥ 65 years who underwent colorectal surgery for CRC at the Tokyo Medical University Hachioji Medical Center between April 2017 and October 2020. Two patients who only underwent stoma creation were excluded (*n* = 2), and the remaining 124 patients were included in this study (Fig. 1). To evaluate atherosclerosis before surgery, these patients underwent the blood pressure/pulse wave test and abdominal unenhanced CT for the calculation of the cardio-ankle vascular index (CAVI) and abdominal aortic calcification (AAC) score, respectively.

The following characteristics of these patients were reviewed: age; sex; American Society of Anesthesiologists (ASA) class; presence of comorbid diseases, such as hypertension, hyperlipidemia, diabetes mellitus, ischemic heart disease, and chronic kidney disease; smoking history; steroid use; stroke; previous abdominal surgery; body mass index; blood test results (albumin and lymphocyte count); prognostic nutritional index [17]; CAVI; AAC score; tumor stage; procedure of colectomy and rectal resection; duration of operation; amount of blood loss; hospital stay; and postoperative complications, including paralytic ileus, intraperitoneal abscess, anastomotic leakage, pneumonia, anastomotic bleeding, and intestinal ischemia.

This study was conducted in accordance with the ethical standards laid down in the 1964 Declaration of Helsinki and its later amendments or comparable ethical standards. The procedures followed were approved by the institutional review board of Tokyo Medical University (T2021-0025). Written informed consent was obtained from all patients prior to their participation in the study.

### Preoperative evaluation of atherosclerosis

The severity of atherosclerosis was evaluated using the blood pressure/pulse wave test and AAC scoring system.

### Blood pressure/pulse wave test

**Fig. 2:**
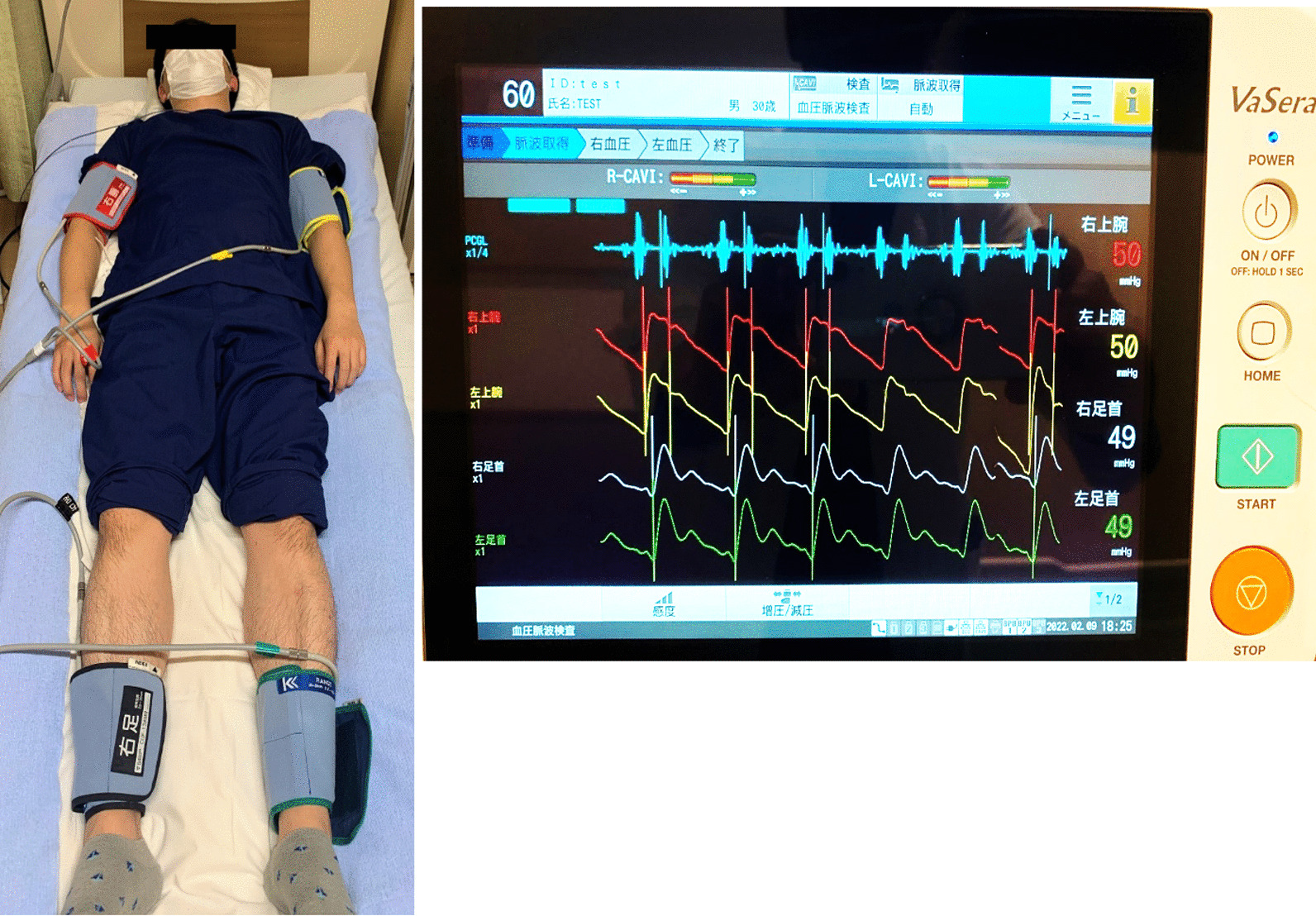
CAVI measurement method. Blood and pulse pressures were determined simultaneously with the patients lying in the supine position (reference image), after they had rested for 10 min in a quiet room

At the time of admission, CAVI was measured using a blood pressure pulse wave analyzer (VaSera VS-2000; Fukuda Denshi Tokyo Nishi Sales Co., Ltd., Tokyo, Japan), according to the method described previously [18]. Briefly, cuffs were applied to the bilateral upper arms and ankles, with the patient lying supine with the head held in the midline position. A microphone was placed on the sternum to monitor the patient’s heartbeat (Fig. 2). Before surgery, the examinations were performed after 10 min of rest in a quiet room. To detect the brachial and ankle pulse waves, inflatable cuffs with the pressure maintained between 30 and 50 mmHg were used to ensure minimal effect of cuff pressure on hemodynamics [19, 20]. Systemic blood and pulse pressure were simultaneously determined. For statistical evaluation of CAVI, mean values of the left and right sides were used. The mean coefficient of the CAVI variation has been shown to be < 5%, which is sufficiently small in clinical usage and indicates that the CAVI measurement has good reproducibility [18].

### AAC scoring system

**Fig. 3:**
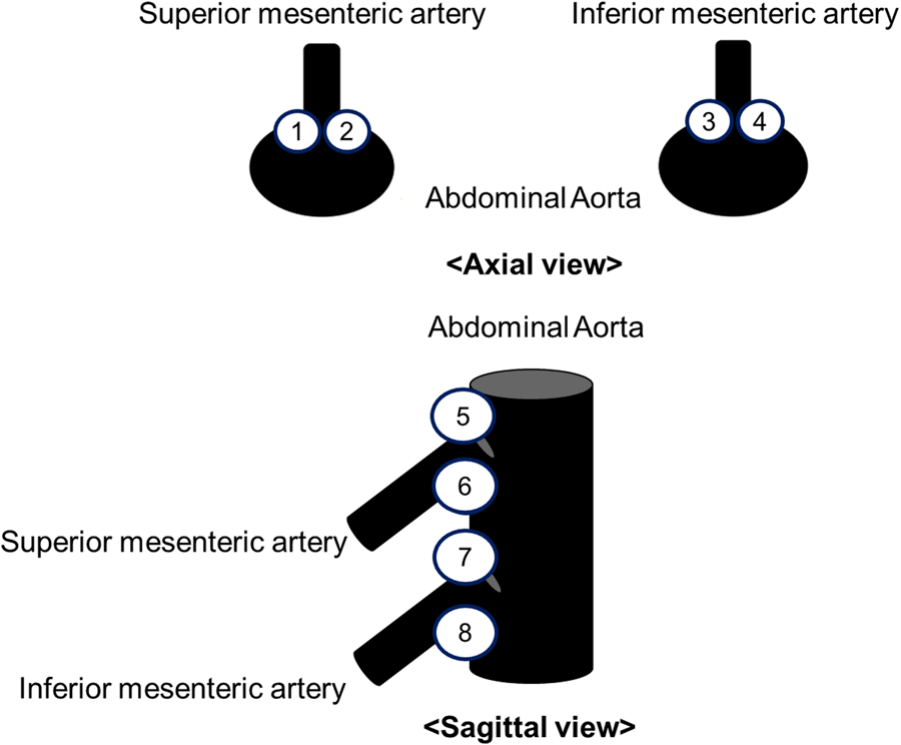
Measurement method for the abdominal aortic calcification (AAC) score. The eight regions of interest around the origin of the superior mesenteric artery and inferior mesenteric artery with a size of 10 mm^2^ were determined. We evaluated the mean density of the areas shown on an unenhanced CT scan

As previously described by Agatston et al. [21], two radiologists in consensus calculated the total number of scores in eight regions around the origin of the superior and inferior mesenteric arteries, which are the major feeders of the colon before surgery. We set the threshold for calcified lesions to a plane CT density of 130 Hounsfield units (HU) and determined a region score based on the mean density in the region of interest size of 10 mm^2^ (1 for 130–199 HU, 2 for 200–299 HU, 3 for 300–399 HU, and 4 for ≥ 400 HU). The total AAC score for each patient was determined by adding the scores from each of the eight regions [21, 22] (Fig. 3).

### Preoperative evaluation of cardiovascular event risk

In our department, we routinely perform echocardiography and electrocardiography to evaluate preoperative cardiac function in surgical patients aged > 65 years under general anesthesia. If there are any cardiovascular diseases related to surgical tolerance, cardiovascular treatment is prioritized before colorectal surgery.

### Postoperative complications

Complications were defined according to the Clavien–Dindo classification scale of I-V. They were grouped as none, minor complications (class I–II), major complications (class III–IV), and death (class V) [23]. We aimed to predict major complications above Clavien–Dindo grade ≥ III because this was indicative of the need for invasive treatment.

### Statistical analyses

All statistical analyses were performed using SPSS software version 27.0 (IBM Corp., Armonk, NY, USA). Categorical variables are expressed as number and percentage (%), whereas continuous variables are presented as mean ± standard deviation. The categorical variables were compared using the chi-squared test or Fisher’s exact test, as appropriate; the continuous variables were compared using the Mann–Whitney U-test. Throughout our analysis, statistical tests were two-tailed, and statistical significance was set at *p*-values of < 0.05. Variables with *p*-values of < 0.25 in the univariate analysis were entered into the multivariate logistic regression model to determine the risk factors for postoperative complications with a criterion of *p* < 0.05. Receiver operating characteristic (ROC) curves were used to determine the cut-off values of CAVI and AAC score. The optimal cut-off value was defined as the point on the ROC curve plotted closest to the point from the upper left corner.

## Results

### Patient characteristics

The study population comprised 124 patients with a mean age of 75.3 ± 6.4 years (Table 1). There were slightly more males (*n* = 76) than females (*n* = 48). Patients with ASA class ≥ 2 accounted for 96.8% of patients, as almost all patients had comorbidities. Overall, the mean AAC score was 7.0 ± 8.0 and the mean CAVI value was 9.5 ± 1.8. Table 2 presents patient- and operation-related factors for postoperative complications. The overall rate of grade ≥ III complications was 11.3% (14 /116), but with no mortality. Postoperative complications (grade ≥ III) included paralytic ileus (*n* = 5), intraperitoneal abscess (*n* = 3), pneumonia (*n* = 2), anastomotic leakage (*n* = 2), intestinal ischemia (*n* = 1), and anastomotic bleeding (*n* = 1).

**Table 1:** Clinicopathological characteristics and surgical outcomes

Variables (*n* = 124)		
*Preoperative information*		
Age	Years	75.3 ± 6.4
Sex	Male	76 (61.3%)
ASA class	1	4 (3.2%)
	2	97 (78.2%)
	≥ 3	23 (18.6%)
Hypertension		64 (51.6%)
Hyperlipidemia		18 (14.5%)
Diabetes mellitus		33 (26.6%)
Ischemic heart disease		18 (14.5%)
Stroke		14 (11.3%)
Chronic renal failure		10 (8.1%)
Smoking history		41 (33.1%)
Past surgical history		33 (26.6%)
Preoperative steroid use		3 (2.4%)
BMI	kg/m^2^	23.0 ± 3.6
Albumin	g/dL	3.7 ± 0.5
Prognostic nutritional index		45.0 ± 5.9
CAVI		9.5 ± 1.8
AAC score		7 ± 8
Stage	I/II	75 (60.5%)
	III/IV	49 (39.5%)
Preoperative chemotherapy		5 (4.0%)
Postoperative chemotherapy		37 (29.8%)
*Surgical information*		
Laparoscopic surgery		102 (82.3%)
Type of surgery	Ileocecal resection	29 (23.4%)
	Right colectomy	24 (19.4%)
	Transverse colectomy	9 (7.3%)
	Left colectomy	7 (5.6%)
	Sigmoidectomy	21 (16.9%)
	Anterior resection	33 (26.6%)
	Miles’/Hartmann’s operation	4 (3.2%)
Operative time	Min	266 ± 119
Estimated blood loss	mL	142 ± 343
*Postoperative information*		
Postoperative hospital stay	Days	16 ± 23
Postoperative complications	Clavien–Dindo grade ≥ III	14 (11.3%)
	Paralytic ileus	5 (4.0%)
	Intraperitoneal abscess	3 (2.4%)
	Anastomotic leakage	2 (1.6%)
	Pneumonia	2 (1.6%)
	Intestinal ischemia	1 (0.8%)
	Anastomotic bleeding	1 (0.8%)

*AAC* abdominal aortic calcification, *ASA* American society of anesthesiologists, *BMI* body mass index, *CAVI* cardio-ankle vascular index

**Table 2:** Predictive factors for postoperative complications

Preoperative variables	Univariate analysis		Multivariate analysis			
	Complication ( −)	Complication ( +)				
	*n* = 110	*n* = 14	*p*-value	Odds ratio	95% CI	*p*-value
*Age*						
years	76.0 ± 6.5	76.5 ± 5.6	0.500			
*Sex*						
male	64	12	0.046			0.633
*ASA class*						
≥ 3	19	4	0.240			0.260
Hypertension	56	8	0.660			
Hyperlipidemia	18	0	0.100			0.151
Diabetes mellitus	29	4	0.540			
Ischemic heart disease	16	2	0.670			
Stroke	12	2	0.490			
Chronic renal failure	7	3	0.090			0.105
Smoking history	36	5	0.520			
Preoperative steroid use	3	0	0.700			
*BMI (kg/m*^*2*^*)*						
< 18, > 25	43	3	0.200			
*Albumin*						
≤ 3 g/dL	9	2	0.360			
*Prognostic nutritional index*						
< 40	20	3	0.500			
Preoperative chemotherapy	4	1	0.460			
AAC score	6 ± 7.6	12 ± 10	0.017	1.083	1.009–1.163	0.026
*Stage*						
I/II	69	6	0.150			0.199
III/IV	41	8				
CAVI	9.4 ± 1.8	10.6 ± 1.5	0.006	1.522	1.073–2.160	0.019
Past surgical history	30	4	0.540			
Laparoscopic surgery	93	9	0.070			0.422
*Type of surgery*						
Ileocecal resection	27	2	0.320			
Right colectomy	21	3	0.540			
Transverse colectomy	9	0	0.330			
Left colectomy	6	1	0.580			
Sigmoidectomy	20	1	0.270			
Anterior resection	27	6	0.130			0.717
Miles’/Hartmann’s operation	3	1	0.390			
*Operative time*						
min	251 ± 97	384 ± 189	0.005	1.007	1.003–1.012	0.001
*Estimated blood loss*						
mL	119 ± 334	325 ± 356	0.076			0.572

*AAC* abdominal aortic calcification, *ASA* American Society of Anesthesiologists, *BMI* body mass index, *CAVI* cardio-ankle vascular index

### Risk factors for postoperative complications

Univariate analysis showed that male sex (*p* = 0.046), AAC score (*p* = 0.017), CAVI value (*p* = 0.006), and operative time (*p* = 0.005) predicted grade ≥ III postoperative complications. The results of logistic regression analysis for postoperative complications are presented in Table 2. The risk factors for postoperative complications were CAVI (odds ratio [OR], 1.522; 95% confidence interval [CI], 1.073–2.160; *p* = 0.019), AAC score (OR, 1.083; 95% CI, 1.009–1.163; *p* = 0.026), and operative time (OR, 1.007; 95% CI, 1.003–1.012; *p* = 0.001).

### Optimal cut-off values of CAVI and AAC score

**Fig. 4:**
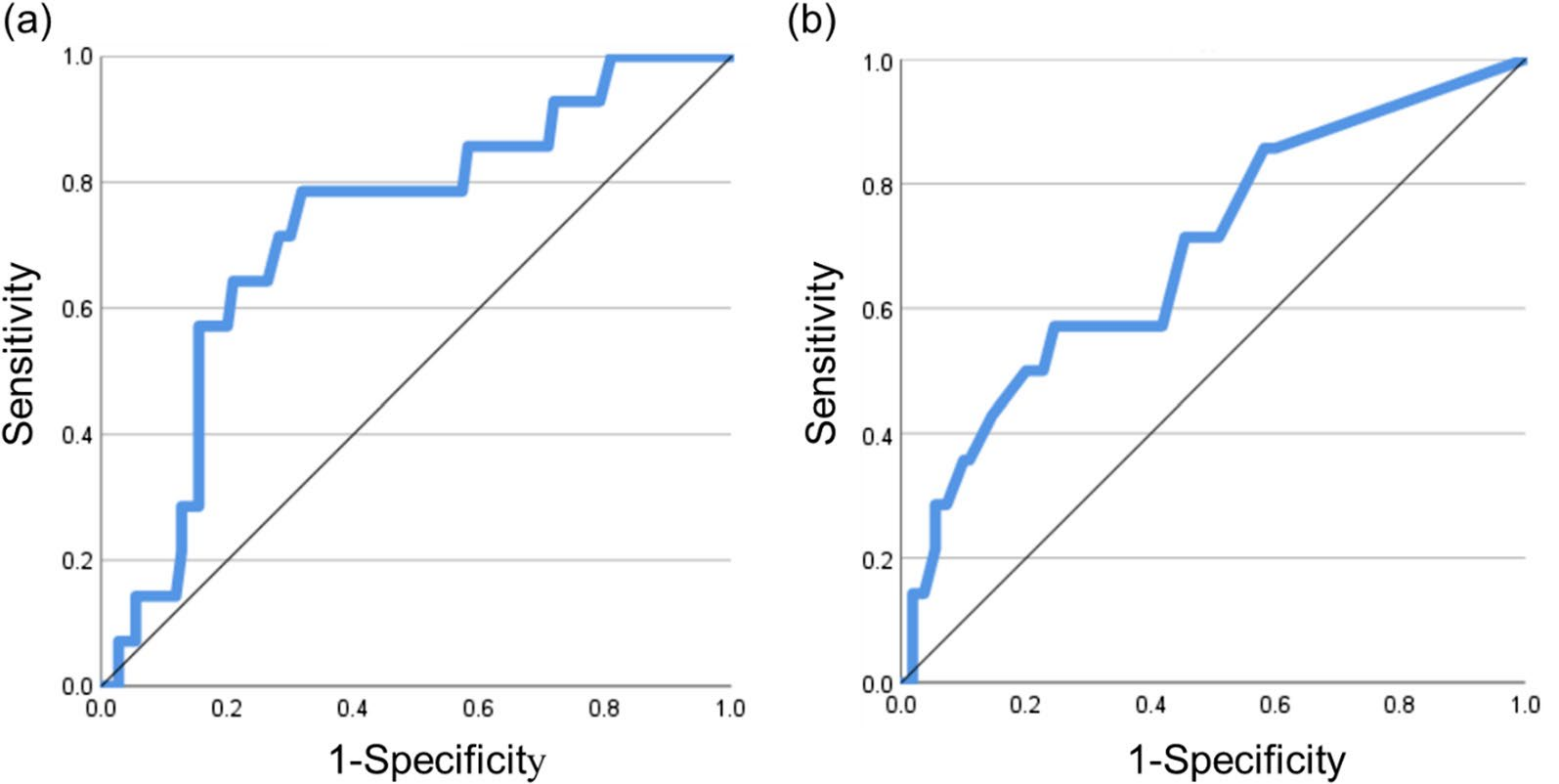
Receiver operating characteristic (ROC) for postoperative complications to set the cut-off values of CAVI and AAC score. **a** The ROC curve indicated that an optimal cut-off value of 10 for CAVI had a sensitivity and specificity of 71.4% and 71.8%, respectively. The area under the ROC curve for CAVI was 0.727 (95% CI, 0.592–0.861). **b** The ROC curve revealed that an optimal cut-off value of 10 for the AAC score had a sensitivity and specificity of 57.1% and 75.5%, respectively. The area under the ROC curve for the AAC score was 0.691 (95% CI, 0.539–0.8441)

The area under the curve was 0.727 and 0.691 for CAVI and AAC score, respectively (Fig. 4). Both CAVI and AAC score had optimal cut-off values of 10.0. Using these cut-off values, the probability of Clavien–Dindo grade ≥ III postoperative complications was compared between the abnormal values of CAVI and AAC score (Table 3). Accordingly, the probability of postoperative complications was 27.8% in patients with abnormal values for both CAVI and AAC score, 20.0% in patients with only abnormal CAVI score, 15.8% in patients with only abnormal AAC score, and 1.6% in patients with normal values for CAVI and AAC score. The incidence of postoperative complications in the group with abnormal values for both CAVI and AAC score was 17.4 times higher than that in the group with normal values for both CAVI and AAC score.

**Table 3:** Probability of postoperative complications (Clavien–Dindo grade ≥ III)

	Complication	Complication	Probability
	( −)	( +)	(%)
CAVI ≥ 10 and AAC score ≥ 10	13	5	27.8
CAVI ≥ 10 and AAC score < 10	20	5	20.0
CAVI < 10 and AAC score ≥ 10	16	3	15.8
CAVI < 10 and AAC score < 10	61	1	1.6

*AAC* abdominal aortic calcification, *CAVI* cardio-ankle vascular index

## Discussion

Generally, risk factors for postoperative complications of colorectal surgery include high ASA score, male sex, poor nutritional status, emergency operations, renal failure, diabetes, anemia, and chronic obstructive pulmonary disease [24]. We previously reported atherosclerosis, male sex, and low skeletal muscle mass as risk factors for postoperative complications in older patients who underwent any type of elective gastrointestinal surgery [25]. The result of the present study showed that atherosclerosis was a risk factor for postoperative complications in older patients with CRC. Atherosclerosis is classified as an age-related disorder, in that increasing age is a risk factor for the development of atherosclerosis [26]. Additionally, atherosclerosis has been reported as the cause of anastomotic leakage in colorectal surgery [15, 16]. However, we demonstrated that not only anastomotic leakage but also other postoperative complications may be associated with atherosclerosis in older patients with CRC. Further, cardiovascular events were not recognized in our study because we evaluated their risk carefully before surgery, as described earlier.

The severity of atherosclerosis was evaluated by CAVI and AAC score in this study. CAVI is a non-invasive test that can objectively evaluate systemic atherosclerosis. It reflects the stiffness of blood vessels from the ascending aorta to the ankle arteries and hence may be a comprehensive marker of systemic atherosclerosis [27]. In addition, CAVI is more comfortable for patients than CT because it does not involve exposure to radiation. Other potential advantages of CAVI include its low estimator dependency and high reproducibility. In line with the manufacturer’s recommendation, CAVI < 8 was considered as normal, 8 ≤ CAVI < 9 as borderline, and CAVI ≥ 9 as abnormal [28]. However, we noted that CAVI increased with age in both sexes [29]. Our study was aimed at older adults; thus, we believed that the high mean CAVI score was a consistent result.

Regarding AAC score, calcification occurs in the media of arteries, and the amount of calcification is directly associated with the extent of atherosclerosis [30]. A previous report has mentioned that AAC score from abdominal nonenhanced CT is a strong predictor of future cardiovascular events [31]. Abdominal CT remains to be the principal staging tool for CRC diagnosis; thus, obtaining information on atherosclerotic calcifications does not require any additional imaging other than CT performed as part of CRC staging [32, 33]. The Agatston score, used in the present study as the calcium score, is the product of the area of calcification (mm^2^) and its radiodensity (HU). It is commonly used in studies of atherosclerosis involving the coronary arteries [15]. Regarding the method of assessing calcification, previous reports have measured different locations such as the aorta and iliac arteries [15, 34, 35]. Moreover, they used either a visual grading system [21, 22] similar to our study or a software-derived calcium score [15, 34, 35]. Accordingly, the assessment of AAC score on CT scan has not yet been appropriately standardized.

CAVI and AAC score evaluate arterial stiffness and arterial calcification, respectively, although both are markers of atherosclerosis. Additionally, in this study, we evaluated the correlation between these two test results and found no significant relationship (see Additional file 1). This suggests that CAVI and AAC score evaluate different aspects of atherosclerosis and that it is useful to perform both tests.

Generally, the postoperative complications of CRC surgery range from paralytic ileus, surgical wound infection, anastomotic leaks and collections, and respiratory infections to impaired renal function. The risk factors for each complication have been investigated; however, cross-sectional studies for all complications have not been adequately conducted. In this study, we examined the relationship between atherosclerosis and all postoperative complications without pre-selection.

The postoperative complications in this study included paralytic ileus, intraperitoneal abscess, and pneumonia. Generally, these complications are considered unrelated to atherosclerosis. However, high CAVI and high AAC score may be associated with paralytic ileus because chronic mesenteric ischemia is typically associated with diffused atherosclerotic disease [36]. Otherwise, the previous study has reported that atherosclerosis is a chronic inflammatory disease, suggesting that chronic infection plays a critical role in the development of atherosclerosis [37]. The infectious complications such as intraperitoneal abscess and pneumonia may accelerate atherosclerosis, causing vascular plaque rupture and decreased tissue perfusion. Moreover, we performed the same analysis of the present study after excluding intraperitoneal abscess and pneumonia from postoperative complications because they may not be related to atherosclerosis. However, CAVI was still a significant factor in both univariate and multivariate analyses (data not shown).

When physicians try to use CAVI and AAC for surgical patients, the atherosclerosis is difficult to be treated preoperatively, even if it is diagnosed by CAVI and AAC. However, high CAVI and high AAC score would be possible to change our surgical techniques involving anastomosis. Firstly, intraoperative indocyanine-green (ICG) angiography has been reported to provide information on tissue perfusion, identifying a well-perfused location for colorectal transections [38]. This is commonly performed in colorectal surgery in Japan and is believed to be useful. The intraoperative ICG of patients with over the ROC cut-off of CAVI and AAC values should be checked. If intraoperative ICG would show delayed tissue perfusion, which would indicate tissue ischemia of the anastomotic site, stoma creation would have to be considered.

Secondly, a previous study has reported that patients with high ligation of the inferior mesenteric artery (IMA above left colic artery) had a 3.8 times higher chance of leaking than those with low ligation (IMA below left colic artery) [39] in sigmoidectomy and anterior resection. Thus, if patients with both abnormal CAVI and AAC score would undergo sigmoidectomy or anterior resection, care should be taken to preserve the left colic artery or superior rectal artery.

Our study had some limitations. First, this was a single-center prospective cohort study; therefore, our findings should be confirmed in a larger-scale study. Second, we only investigated calcification around the origin of the superior mesenteric artery and inferior mesenteric artery based on the AAC score. However, to assess systemic atherosclerosis, other regions may have to be investigated as well.

## Conclusions

Atherosclerosis, evaluated based on CAVI and AAC score, was a risk factor for postoperative complications of elective colorectal surgery in older patients with CRC. We emphasize that the preoperative evaluations of atherosclerosis in colorectal surgery in older patients with CRC may help surgeons perform surgery safely and appropriately tailor postoperative management.

## Supplementary Information

**Additional file 1**: Scatter plots of CAVI and AAC score. Pearson's correlation coefficient was 0.10 between AAC score and CAVI

## Data Availability

All data related to this study and any pertinent analysis are available upon request. Requests should be directed to the corresponding author.

## Abbreviations

CRC: Colorectal cancer
CAVI: Cardio-ankle vascular index
AAC: Abdominal aortic calcification
CT: Computed tomography
ASA: American Society of Anesthesiologists
HU: Hounsfield units
ROC: Receiver operating characteristic
