# Supplementary figures and images for: Impact of atherosclerosis on the postoperative complications of colorectal surgery in older patients with colorectal cancer
Source: BMC Gastroenterol. 2022 Dec 14;22:519. doi: 10.1186/s12876-022-02600-7 (PMC9749351; doi:10.1186/s12876-022-02600-7)

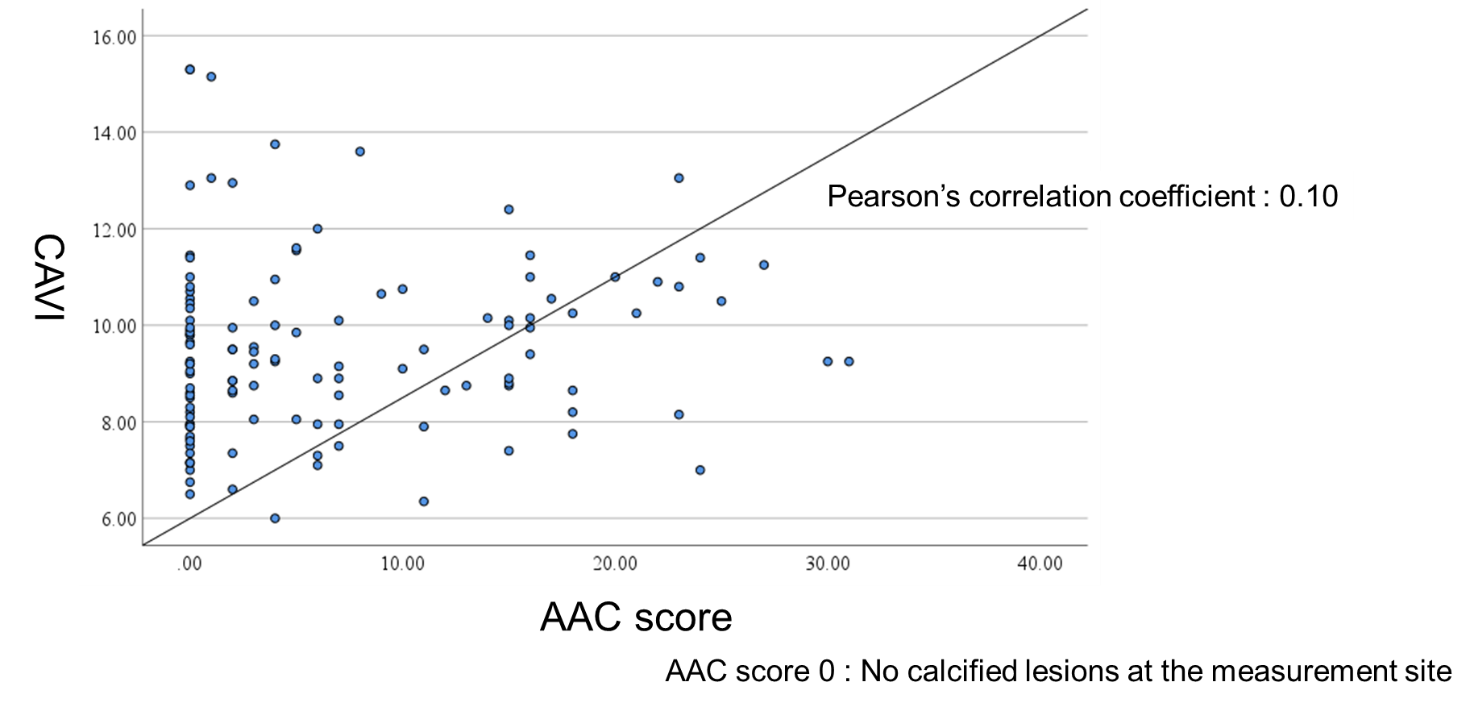


**Additional File 1** Scatter plots of CAVI and AAC score.

Supplement: Supplementary file 1 — Additional file 1: Scatter plots of CAVI and AAC score. Pearson's correlation coefficient was 0.10 between AAC score and CAVI [file 12876_2022_2600_MOESM1_ESM.docx]
